# Dynamics of Infections in Cattle and *Rhipicephalus microplus*: A Preliminary Study

**DOI:** 10.3390/pathogens12080998

**Published:** 2023-07-30

**Authors:** Elianne Piloto-Sardiñas, Angélique Foucault-Simonin, Alejandra Wu-Chuang, Lourdes Mateos-Hernández, Roxana Marrero-Perera, Lianet Abuin-Denis, Lisset Roblejo-Arias, Cristian Díaz-Corona, Zbigniew Zając, Joanna Kulisz, Aneta Woźniak, Sara Moutailler, Belkis Corona-González, Alejandro Cabezas-Cruz

**Affiliations:** 1 ANSES, INRAE, Ecole Nationale Vétérinaire d’Alfort, UMR BIPAR, Laboratoire de Santé Animale, Maisons-Alfort 94700, France; elianne9409@gmail.com (E.P.-S.); angelique.foucaultsimonin@anses.fr (A.F.-S.); alewch29@gmail.com (A.W.-C.); lourdes.mateos@vet-alfort.fr (L.M.-H.); labuind@gmail.com (L.A.-D.);; 2 Direction of Animal Health, National Center for Animal and Plant Health, Carretera de Tapaste y Autopista Nacional, Apartado Postal 10, San José de las Lajas 32700, Mayabeque, Cuba; roxanamarrerop95@gmail.com (R.M.-P.); lroblejoarias90@gmail.com (L.R.-A.); cristiandiaz9603@gmail.com (C.D.-C.); 3 Department of Biology and Parasitology, Medical University of Lublin, Radziwiłłowska 11 St., 20-080 Lublin, Poland; zbigniew.zajac@umlub.pl (Z.Z.); joanna.kulisz@umlub.pl (J.K.); aneta.wozniak@umlub.pl (A.W.)

**Keywords:** dynamics, tick-borne pathogens, cattle, ticks, real-time microfluidic PCR

## Abstract

Tick-borne pathogens (TBPs) pose a significant threat to livestock, including bovine species. This study aimed to investigate TBPs in cattle and ticks across four sampling points, utilizing real-time microfluidic PCR. The results revealed that *Rhipicephalus microplus* ticks were found infesting all animals. Among the detected TBPs in cattle, *Anaplasma marginale* was the most frequently identified, often as a single infection, although mixed infections involving *Rickettsia felis*, uncharacterized *Rickettsia* sp., and *Anaplasma* sp. were also observed. In ticks, *A. marginale* was predominant, along with *R. felis*, *Rickettsia* sp., and *Ehrlichia* sp. It is noteworthy that although *A. marginale* consistently infected all cattle during various sampling times, this pathogen was not detected in all ticks. This suggests a complex dynamic of pathogen acquisition by ticks. A phylogenetic analysis focused on the identification of *Anaplasma* species using amplified 16S rDNA gene fragments revealed the presence of *A. marginale* and *Anaplasma platys* strains in bovines. These findings underscore the presence of multiple TBPs in both cattle and ticks, with *A. marginale* being the most prevalent. Understanding the dynamics and phylogenetics of TBPs is crucial for developing effective control strategies to mitigate tick-borne diseases in livestock.

## 1. Introduction

Tick-borne pathogens (TBPs) are a significant problem globally, particularly in tropical and subtropical regions, affecting livestock industries and animal health [1,2]. The main pathogens responsible for productivity loss and health issues in livestock are tick-borne protozoa, namely *Babesia* (*B. bovis* and *B. bigemina*) and *Theileria* (*T. annulata*, *T. sinensis*, and *T. orientalis*), as well as tick-borne rickettsial pathogens, such as *Anaplasma* (*A. marginale*, *A. bovis*, *A. centrale*, *A. phagocytophilum*, and *A. platys*) and *Ehrlichia* (*E. ruminantium* and *E. minasensis*) [1,3,4].

In Cuba, the major pathogens affecting cattle are *B. bovis*, *B. bigemina*, and *A. marginale*, which cause diseases known as babesiosis and anaplasmosis. These diseases collectively form a complex called cattle tick fever (CTF), and the primary vector for these pathogens is *Rhipicephalus microplus* [5,6]. The climate conditions in Cuba, characterized by high temperature and relative humidity, promote the survival of *R. microplus* and infestation in cattle, making tick eradication challenging [6]. The presence of pathogens, host susceptibility, increased tick populations, and subsequent infestation all contribute to the transmission of these pathogens [7].

Bovine anaplasmosis affects cattle of all ages, with increased susceptibility and disease development as animals age [8]. Clinical signs include anorexia, jaundice, abortion, weight loss, reduced meat and milk production, and in severe cases, even death [9]. *Anaplasma marginale* infection is persistent, resulting in carrier animals. Diagnosis of the infection can be conducted through direct pathogen detection using blood smears or serological methods [8]. However, due to low parasitemia levels during persistent infection and potential cross-reactivity with other species, molecular techniques such as PCR variants are necessary. Similarly, during bovine babesiosis, persistent infection occurs in animals that survive the acute phase [5]. The clinical signs of infection caused by *B. bigemina* are generally less severe, although the severity can vary depending on the region due to the variability of the virulence factors of the parasites [10]. Infections by *B. bigemina* and *B. bovis* result in hemoglobinuria, jaundice, and anemia [10]. Microscopic techniques are suitable for detecting *B. bovis* and *B. bigemina*; however, their low sensitivity limits their use in epidemiological studies to detect carrier animals [11]. Serological methods have been used, but they face challenges due to cross-reactivity among species and the inability to differentiate between past and recent infections [11]. Thus, PCR-based tests provide high sensitivity and specificity for detecting pathogens in both mixed and simple infections.

Considering the significant threat of TBP diseases to livestock populations and their zoonotic potential, reliable and rapid identification of the causative agents is crucial [12,13,14]. Although PCR-based molecular techniques offer high sensitivity and specificity, they have limitations, such as the inability to detect numerous pathogens simultaneously, the requirement for large volumes of nucleic acids, and the lack of targeting commensals or endosymbionts. To overcome these limitations, microfluidic-based techniques, a variant of real-time PCR, are employed. High-throughput microfluidic methods allow the simultaneous detection of pathogens and are widely used in epidemiological and surveillance studies. In this study, our objective was to employ real-time microfluidic PCR for the simultaneous detection of TBPs with different etiologies in cattle and ticks. We also aimed to analyze the infection patterns over time, with a specific focus on *A. marginale* based on previous reports of its prevalence in Cuban bovines.

## 2. Materials and Methods

### 2.1. Study Design

A longitudinal study was conducted on a farm in Mayabeque province, Cuba, spanning from March 2020 to March 2021. The study focused on eight female bovines randomly selected at the beginning of the research. Samples were taken at various times during both the dry and wet climatic periods, ensuring two samples from each period, resulting in a total of 32 bovine samples. All the animals belonged to the Siboney breed and were older than 2 years of age. They were raised extensively on pasture without any acaricide treatment. Engorged female ticks were collected from infested animals in July and September 2020, as well as in March 2021. Throughout the study, all animals remained asymptomatic. During the first and second sampling times, hematological parameters, including hematocrit, total leukocyte count, and total protein, were determined to assess the health status of the investigated animals.

### 2.2. Blood Samples Collection and Haematological Parameters

Samples were collected from the jugular vein using sterile Vacutainer needles and K2EDTA-coated tubes (Becton-Dickinson Vacutainer Systems, Franklin Lakes, NJ, USA) and stored at 4 °C. The hemogram analysis included the evaluation of hematocrit (HCT) and total white blood cell counts (WBCs). HCT was determined by microcentrifugation of each blood sample using a Jouan Hema-C microhematocrit centrifuge (Hawksley and Sons, Ltd., Sussex, UK) at 18,600× *g* for 5 min. The HCT value was measured using a DAMON/IEC hematocrit reader (Damon/IEC Division, Needham Heights, MA, USA). Normal values were considered to be in the range of 0.27 L/L to 0.47 L/L [15].

WBC counts were performed using a Neubauer chamber, employing 2% acetic acid and 1/20 diluted blood. Normal values for WBCs were in the range of 4.0–12.0 (c/L) [16,17]. Additionally, the concentration of total protein in the liquid portion of the blood was determined using a portable refractometer (Fisher Scientific, F67403 Illkirch Cedex, France). Concentration values ranging from 56.9 g/L to 78.7 g/L were considered normal [18].

### 2.3. Tick Collection and Morphological Identification

All collected specimens were carefully placed in labeled plastic tubes. To ensure their survival during transportation, each tube was covered with a piece of cloth and secured with a rubber band. The live ticks were then transported to the laboratory for further analysis. In the laboratory, the ticks were identified using a stereomicroscope (Carl Zeiss AG, Oberkochen, Germany), following the standardized taxonomic keys described by Estrada-Peña et al. [19]. Once identified, the ticks were preserved in 70% ethanol (Merck^®^, Kenilworth, NJ, USA) within sterile 1.5 mL plastic tubes. These tubes were stored at −80 °C until DNA extraction could be performed.

### 2.4. Blood and Tick DNA Extraction

DNA extraction from blood samples was conducted within 24 h of collection using the Wizard Genomic DNA Purification Kit (Promega, Madison, WI, USA) following the manufacturer’s instructions. The DNA samples were eluted in 100 µL of DNA Rehydration Solution and stored at −80 °C until they were used as templates for polymerase chain reaction (PCR) assays.

For tick samples, one female tick per animal was selected from the total collected during the last three sampling sessions for total DNA extraction. Ticks were homogenized using a MagNA Lyser instrument (Roche Molecular Diagnostics, Rotkreuz, Switzerland) at a speed of 5000 rpm for 5 cycles of 60 s each. During homogenization, 50 μL of PBS (Sigma, St. Louis, MO, USA) were added to a MagNA Lyser tube containing ceramic beads. The tick homogenates were then subjected to total DNA extraction using the Wizard Genomic DNA Purification kit (Promega, Madison, WI, USA) according to the manufacturer’s instructions. The DNA samples were eluted in 60 µL of DNA Rehydration Solution. The quantitative and qualitative assessment of DNA extraction was performed using a Colibri Microvolume Spectrophotometer (Titertek-Berthold, Pforzheim, Germany). All extracted DNA samples were stored at −80 °C until they were used.

### 2.5. Molecular Detection of Tick-Borne Pathogens

#### 2.5.1. DNA Pre-Amplification for Real-Time Microfluidic PCR

To enhance the detection of pathogen DNA, the total DNA underwent pre-amplification using the PreAmp Master Mix (Standard Biotools, CA, USA) following the manufacturer’s instructions. Primers, except those targeting tick DNA and controls, were combined in equal volumes to create a pooled primer mix with a final concentration of 200 nM. The reaction was carried out in a 5 μL final volume, containing 1 μL of Perfecta Preamp 5×, 1.25 μL of the pooled primer mix, 1.5 μL of distilled water, and 1.25 μL of DNA. The thermocycling program included an initial cycle at 95 °C for 2 min, followed by 14 cycles at 95 °C for 15 s and 60 °C for 4 min. After completing the cycling program, the reactions were diluted 1:10 in Milli-Q ultrapure water. All pre-amplified DNA samples were stored at −20 °C until further use.

#### 2.5.2. Real-Time Microfluidic PCR

The presence of major tick-borne pathogens (TBPs), endosymbionts, parasite species, bacterial genera, and parasite taxa was evaluated using high-throughput real-time microfluidic PCR amplification. The assessment utilized 48.48 Dynamic Array™ IFC chips from Standard Biotools (Fluidigm, South San Francisco, CA, USA) and the BioMark™ real-time PCR system. These chips facilitated the dispensing of 48 PCR mixes and 48 samples into individual wells. On-chip microfluidics then assembled real-time PCR reactions in separate chambers before thermal cycling, resulting in 2304 individual reactions. TaqMan Gene expression master mix from Applied Biosystems (Courtaboeuf, France) was used along with 6-carboxyfluorescein (FAM) and black hole quencher (BHQ1)-labeled TaqMan probes, following the manufacturer’s instructions.

The PCR cycling protocol consisted of 2 min at 50 °C, followed by 10 min at 95 °C. This was followed by 40 cycles of two-step amplification: 15 s at 95 °C and 1 min at 60 °C. To account for potential PCR inhibition, an *Escherichia coli* strain EDL933 DNA was added as an internal control to each sample. Primers and a probe specific to the *E. coli* gene were utilized. Additionally, a negative water control was included on each chip.

The high-throughput real-time microfluidic PCR method allowed for the detection of 27 bacterial species (including *Borrelia*, *Anaplasma*, *Ehrlichia*, *Rickettsia*, and *Mycoplasma*), 7 parasite species (such as *Babesia* and *Hepatozoon*), 5 bacterial genera, and 3 parasite taxa (Apicomplexa, *Theileria*, and *Hepatozoon*). Supplementary Table S1 [20] contains information about the target genes and primer sequences used for amplification. The development of this new high-performance tool, based on real-time microfluidic PCR, involved several crucial elements: sensitivity testing, specificity evaluation, and the implementation of essential controls. Grech-Angelini et al. [21] and Michelet et al. [22] have provided detailed descriptions of these aspects in their research. The use of this technique provided an efficient and comprehensive assessment of the presence of these pathogens and endosymbionts.

#### 2.5.3. PCR and DNA Sequencing for Anaplasma Species Identification

To validate the results obtained from real-time microfluidic PCR, a subset of positive samples for *Anaplasma* spp. were selected for further analysis using conventional and nested PCR assays by amplification of 16S rDNA gene. These additional assays employed primers different from those utilized in the BioMark^TM^ system [23].

For amplicon sequencing, Eurofins MWG Operon (Ebersberg, Germany) was commissioned, and the obtained sequences were assembled using BioEdit 7.2 software (Ibis Biosciences, Carlsbad, CA, USA). The resulting nucleotide sequences were then compared against the GenBank database using the Basic Local Alignment Sequence Tool (BLAST) search engine1 of the National Center for Biotechnology Information (NCBI; Bethesda, MD, USA). Accession numbers OQ362275-OQ362281 and OQ619389-OQ619392 were assigned to the nucleotide sequence data reported in this study. These data are available in the GenBank, EMBL, and DDBJ databases.

### 2.6. Phylogenetic Analysis

In this study, the phylogenetic relationship of *Anaplasma* spp. sequences was inferred using the 16S rDNA gene. The obtained sequences were compared to the NCBI GenBank database (https://blast.ncbi.nlm.nih.gov/blast.cgi, accessed on 30 January 2023) through a BLAST search. Sequences representing all continents, if available, and showing similarity to our samples were selected for further analysis. At least three sequences for each *Anaplasma* species were included.

The sequences were aligned using the Muscle algorithm in MEGA 11 software. To construct phylograms, three methods were utilized: maximum parsimony (MP), neighbor-joining (NJ), and maximum likelihood (ML). Since the ML method and the other two methods showed similar topologies, the ML method was chosen for the final analysis. The selection of the appropriate model for tree construction was based on the lowest Bayesian information criterion (BIC) and corrected Akaike information criterion (AICc). The Kimura 2-parameter model (K2), with the removal of unaligned positions using complete deletion, was used to build the tree. To assess the reliability of internal branches, the bootstrapping method with 300 replicates was employed [24].

## 3. Results

### 3.1. Bovine Hematological Parameters and Taxonomic Identification of Collected Ticks

All animals exhibited hematocrit and total protein values within the normal physiological range for bovine species during the two sampling times analyzed. However, at the beginning of the study, four animals showed elevated blood leukocyte levels (Table 1).

Additionally, all animals were found to be infested with ticks. A total of 350 adult ticks (153 females and 197 males) were morphologically identified as *Rhipicephalus microplus*.

### 3.2. Detection of TBPs in Cattle Samples and Ticks

The study revealed the presence of several tick-borne pathogens (TBPs) in the blood samples. *Anaplasma marginale* was detected in 31 out of 32 samples (96.9%; 95% CI: 90.9–100), followed by *Rickettsia felis* in four samples (12.5%; 95% CI: 1.10–23.9), uncharacterized bacteria within the *Rickettsia* genus in two samples (6.25%; 95% CI: 0–7.09), and *Anaplasma* spp. in one sample (3.12%; 95% CI: 0–9.19) (Table 2). *Anaplasma marginale* was the sole pathogen detected in 26 out of the total samples (81.3%; 95% CI: 67.8–94.8) as a single infection. Mixed infections were found in six samples, with *R. felis* + *A. marginale* being the most common co-infection (3/32, 9.38%; 95% CI: 0–19.5), followed by *A. marginale* + *Rickettsia* spp. (2/32, 6.25%; 95% CI: 0–7.09), and *R. felis* + *Anaplasma* spp. (1/32, 3.13%; 95% CI: 0–0.11).

To detect the presence of TBPs in ticks, one female tick per animal was selected during each sampling period, resulting in a total of 24 ticks being analyzed. Among these ticks, *A. marginale* DNA was detected in 11 (45.8%; 95% CI:25.9–65.7–64.4), followed by *R. felis* in four ticks (16.7%; 95% CI: 1.81– 31.5), *Ehrlichia* spp. in two ticks (8.33%; 95% CI: 2.69–13.9), and *Rickettsia* spp. in one tick (4.17%; 95% CI: 0.1–8.24) (Table 3). In terms of single infections, *A. marginale* was the most common TBP detected in tick with eight ticks (33.3%; 95% CI: 14.2–51.8), followed by *R. felis* in three ticks (12.5%; 95% CI: 5.75–19.3), and *Ehrlichia* spp. in one tick (4.17%; 95% CI: 0.1–8.24). *Anaplasma marginale* was detected in double infection with *R. felis* and *Rickettsia* spp. and in triple infection with *Ehrlichia* spp. and *B. canis* (subspecies) (Table 3).

### 3.3. Dynamics of Tick-Borne Pathogen Infection in Cattle and Ticks

**Figure 1:**
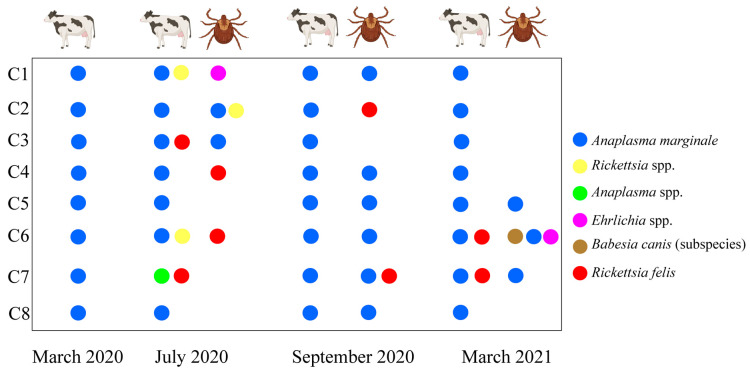
Dynamics of vector-borne rickettsial pathogens detected in cattle and ticks by real-time microfluidic PCR.

All animals tested positive for *A. marginale* throughout the four time points, except in animal C7 where only *Anaplasma* spp. at the genus level could be detected. Infections with other pathogens, such as *Rickettsia* spp. and *R. felis,* were not persistent over time. However, in animal C7, *R. felis* was detected in the July 2020 and March 2021 samples (Figure 1).

During the corresponding sampling periods, *A. marginale* was identified in both ticks and the respective animals from which they were collected. However, *Ehrlichia* spp., *R. felis*, and *Rickettsia* spp. were detected exclusively in ticks and not in the sampled animals from which the ticks were collected. Notably, *A. marginale* was the only pathogen observed in ticks collected from the same animal during different sampling times (Figure 1).

### 3.4. Phylogenetic Analysis of Identified Anaplasma Species

**Figure 2:**
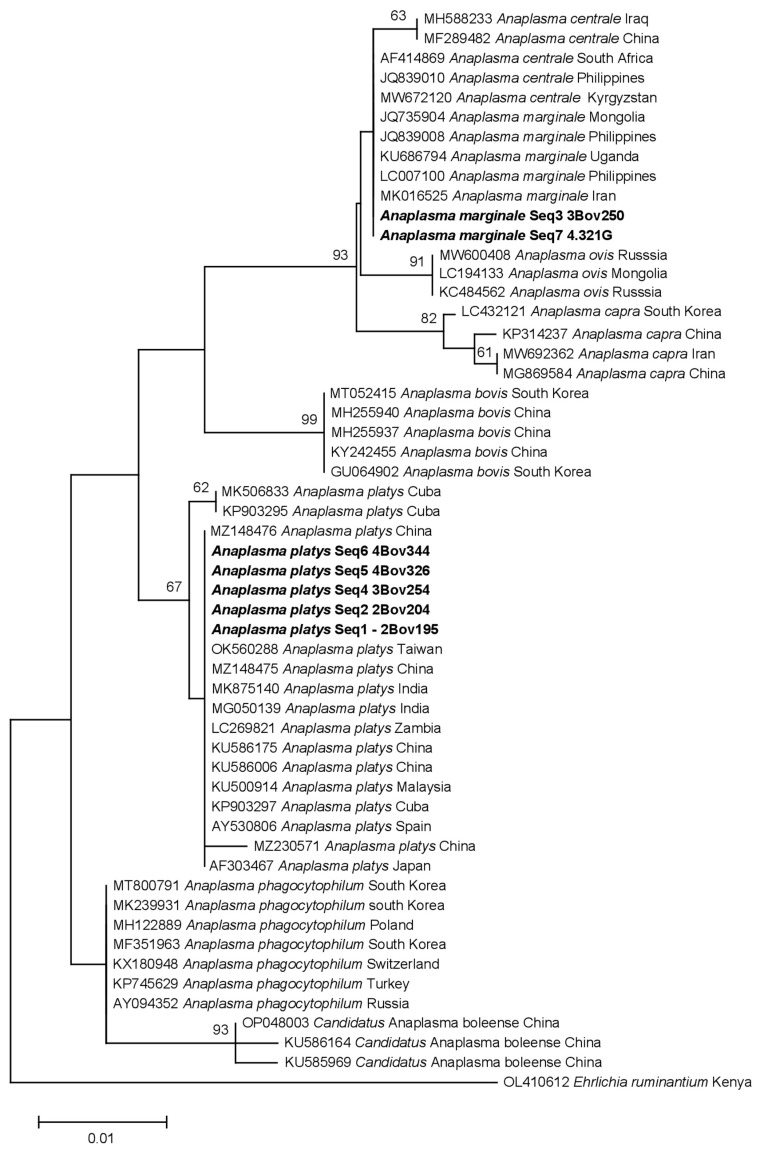
Phylogeny of *Anaplasma* spp. inferred from 16S rDNA. The evolutionary history was inferred using the maximum likelihood method and the Kimura 2-parameter model. The analysis contains *Anaplasma* spp. 16S rDNA sequences identified in the current study (bold) and GenBank sequences. Accession numbers of sequences are given. Bootstrap values are represented as per cent of internal branches (300 replicates); values lower than 60 are hidden. The tree is drawn to scale, with branch lengths measured in the number of substitutions per site. This analysis involved 55 nucleotide sequences. There were a total of 301 positions in the final dataset. Sequence OL410612 *Ehrlichia ruminantium* was used to root the tree.

By blasting the obtained sequences (16S rDNA) against the NCBI GenBank database, we found that they exhibited similarity to various *Anaplasma* species, including *A. bovis*, *A. capra*, *A. centrale*, *A. marginale*, *A. ovis*, *A. phagocytophilum*, *A. platys*, and *Candidatus* Anaplasma boleense. Specifically, Seq3 3Bov250 (OQ362277) and Seq7 4.321G (OQ362281) were identified as *A. marginale* and were grouped together with *A. marginale* sequences from Mongolia (JQ735904), Philippines (LC007100), and Uganda (KU686794), as well as *A. centrale* sequences from South Africa (AF414869) and Kyrgyzstan (MW672120) (Figure 2). On the other hand, Seq1-2Bov195 (OQ362275), Seq2 2Bov204 (OQ362276), Seq4 3Bov254 (OQ362278), Seq5 4Bov326 (OQ362279), and Seq6 4Bov344 (OQ362280) were clustered with *A. platys* sequences reported from various locations, such as Cuba (MK506833), India (MK875140), Zambia (LC269821), and Spain (AY530806).

## 4. Discussion

This study focused on investigating tick-borne pathogens (TBPs) in ticks and cattle over time, particularly examining hematological parameters and molecular detection of TBPs using real-time microfluidic PCR. As a preliminary and observational study, it aimed to generate initial insights and identify potential associations between TBPs and cattle health. We acknowledge the absence of statistical analysis in this study, which is primarily due to the exploratory nature of the research and the small sample size of asymptomatic animals. While statistical tests could provide valuable information, our focus was on qualitative observations and descriptive data to identify patterns and generate hypotheses for future investigations.

Hematological parameters were analyzed during the first and second sampling times, and it was found that hematocrit and total protein values in all cases fell within the normal range for bovine species, although some animals showed leukocytosis at the beginning of the study. Hematological parameters and serum total protein concentrations can be influenced by genetic factors (such as breed and genotype) and non-genetic factors (including pathogen infection, age, sex, management system, medication, health status, and environmental factors) [25]. Monitoring these parameters and comparing them with reference values provide insights into the prognosis of diseases, infections, and the overall health status of animals, as changes in these parameters indicate pathophysiological responses [26]. The sampled animals in this study were asymptomatic, and the maintenance of values within the normal range indicated good health.

Among the TBPs detected using real-time microfluidic PCR, *A. marginale* was the most frequent pathogen. This pathogen was identified in the same animals during different sampling periods and in ticks collected from the same animals at different times. *Anaplasma marginale* can persist in cattle herds without causing clinical signs after an acute anaplasmosis phase [27]. In these animals, rickettsemia occurs periodically, with parasitemia levels often falling below the detection threshold of light microscopy [28]. These asymptomatic animals act as reservoirs within herds, highlighting the importance of efficient and rapid pathogen detection to prevent its spread to non-infected animals. Molecular detection of *A. marginale* using PCR variants is a fast and effective diagnostic tool, especially in asymptomatic carriers with low bacteremia levels [28,29,30]. Conventional PCR-based diagnostic methods have limitations, such as the inability to detect multiple pathogens simultaneously and the requirement for larger volumes of nucleic acid, which can be overcome using real-time microfluidic PCR [31]. In our study, real-time microfluidic PCR enabled the detection of *A. marginale* in both animals and ticks across all four sampling periods, demonstrating the persistent infection of *A. marginale* in asymptomatic cattle over time, the role of these animals as reservoirs for chronic infections, the ability of *R. microplus* to acquire pathogens even at low concentrations in the host, and the sensitivity of molecular methods, particularly real-time microfluidic PCR, in detecting TBPs in asymptomatic animals.

*Rhipicephalus microplus* is a single-host tick that exhibits slow and fast feeding phases during its parasitic stage on the host’s body [32]. The tick’s adherence to the host and the feeding process can facilitate the acquisition of potentially pathogenic or non-pathogenic microorganisms. Despite the persistent infection by *A. marginale* in the host, the adherence of *R. microplus* during all its life stages, and the use of a molecular detection technique with high specificity and sensitivity, *A. marginale* was not detected in some of the ticks analyzed. The lack of infection of engorged adult ticks on persistently infected cattle can be attributed to a combination of factors. Firstly, a low microbial load during varying rickettsemia levels due to the cyclical appearance of antigenic variants in persistent infection might reduce the probability of successful transmission to feeding ticks [33,34]. Secondly, negative interactions between *A. marginale* and other microorganisms coexisting in the tick-feeding site could hinder the acquisition process of *A. marginale* by *R. microplus* [30]. Studies carried out on other species of ticks show that low levels of parasitemia hinder the tick’s ability to acquire the pathogen [35]. In some cases, despite a low acquisition capacity, the replication of the microorganism in a tick is capable of compensating for the differences in the initial infection dose [35]; however, other factors would come into play within the vector. Tick immunity or resistance to *A. marginale*, along with variations in tick developmental stages and environmental conditions, may also play a role in preventing infection [36]. Additionally, in the host, the development of a robust humoral and cellular immune response in persistently infected cattle could interfere with tick fitness and influence vector competition, limiting the ability of ticks to acquire the pathogen [37,38,39]. Understanding these multifaceted factors is crucial for gaining insights into the transmission dynamics of tick-borne pathogens and their interactions with hosts. Further research is warranted to fully elucidate the mechanisms involved in this intriguing phenomenon.

Mixed infections involving *A. marginale*, rickettsial pathogens (*R. felis*, *Rickettsia* spp., *Anaplasma* spp., and *Ehrlichia* spp.), and protozoa (*B. canis*) were also detected. Previous studies have reported mixed infections in cattle, such as *A. marginale,* with rickettsial pathogens such as *A. phagocytophilum*, *A. centrale*, and *A. bovis* [40,41,42], and protozoa such as *B. bigemina* and *T. annulata* [43,44]. During mixed infections, positive interactions between pathogens can enhance their multiplication and colonization, facilitating disease transmission and progression in hosts. Mixed infections have the potential to exacerbate clinical symptoms, but certain studies propose that they could also confer benefits to animals. Less pathogenic species within mixed infections may stimulate the host’s immune system, leading to the development of immunity against other species with varying pathogenicity, a phenomenon known as heterologous protection [45,46]. Although mixed infections were detected in some animals in the present study, all animals remained asymptomatic throughout the study. Based on these findings, it can be hypothesized that the absence of symptoms may be due to negative interactions between microorganisms, where the presence of one suppresses the virulence or colonization of others, or it may be attributed to the phenomenon of heterologous immunity.

Another important finding of the study is the detection of *R. felis* in cattle, and *R. microplus* ticks. *Rickettsia felis* is considered to have a cosmopolitan distribution [47] from an ecological perspective. It is typically transmitted by *Ctenocephalides felis* [48], but it has been detected in other species of fleas, ticks, and mites, suggesting a possible role of other arthropods as hosts and vectors, and it is also known for its zoonotic potential [49]. Its presence has been previously reported in cattle [50] and in *R. microplus* ticks [51]. Additionally, in Cuba, Diaz et al. [52] detected it in *Dermacentor nitens* ticks infesting horses. Our results constitute the first molecular evidence of *R. felis* in Cuban cattle, and *R. microplus* ticks, suggesting a possible broad vertebrate and arthropod host range.

Phylogenetic analysis, focused on the identification of *Anaplasma*, confirmed the presence of *A. marginale* and *A. platys* in the animals. *Anaplasma platys* is the causative agent of canine infectious cyclic thrombocytopenia in dogs and is typically transmitted by *Rhipicephalus sanguineus* sensu lato ticks [53,54,55]. However, the presence of *A. platys* has also been reported in cattle in different parts of the world, such as Algeria and Egypt, and co-infections with *A. marginale* have been documented in cattle [56,57,58,59]. Infection by *A. marginale* has been previously reported in Cuban water buffalo [7,60] and cattle [61], while *A. platys* has been detected in dogs [62,63]. However, this study provides the first molecular evidence of *A. platys* infection in *R. microplus* and cattle and the first report of mixed infection between *A. marginale* and *A. platys* in Cuban cattle. These findings are significant not only for animal health but also for human health, as the zoonotic potential of *A. platys* has been documented [64]. The presence of *A. platys* in cattle gains even more importance when we consider the characteristics of our scenario. Neither dogs nor cats were present in the system, and none of the collected ticks were identified as *R. sanguineus* based on their morphology. Hence, there is no concrete evidence to support the idea that the detected *A. platys* infection in cattle is due to an infestation of cattle by *R. sanguineus*. Given the above information, we can hypothesize that the detection of *A. platys* in cattle suggests its possible involvement as a causative agent of bovine anaplasmosis, along with *A. marginale*. Additionally, this raises the possibility that *R. microplus* may play a role in the transmission of *A. platys*. These implications are crucial for understanding and managing the health of both animals and humans in the region.

## 5. Conclusions

This preliminary study investigated the simultaneous detection and dynamics of TBPs in healthy cattle and ticks in a specific region by the use of real-time microfluidic PCR. *Anaplasma marginale* was the most common pathogen detected in both cattle samples and ticks. The occurrence of single and mixed infections was observed, with co-infection of *R. felis* and *A. marginale* being the most common. The presence of TBPs in ticks further highlights their role as potential vectors for transmitting these pathogens. The dynamics of TBP infections in both cattle and ticks were examined over four sampling time points. *Anaplasma marginale* was consistently detected in all cattle samples throughout the study, indicating its persistent presence. In contrast, infections with *Rickettsia* spp. and *R. felis* were not persistent over time. Phylogenetic analysis of the identified *Anaplasma* species revealed similarities to various species, including *A. marginale* and *A. platys*. The sequences clustered with strains from different geographical locations, suggesting a wide distribution of these pathogens. These findings emphasize the importance of monitoring and understanding the occurrence and dynamics of TBPs in cattle and ticks. The presence of these pathogens poses a potential threat to livestock health and highlights the need for effective control and prevention strategies. Further studies are warranted to investigate the transmission dynamics and assess the impact of these infections on animal health and productivity.

## Figures and Tables

**Table 1:** Hematological parameters measured in animal blood during the first and second sampling times.

Animals ID	HCT * 0.27–0.47 (L/L)		WBCs * 4.0–12.0 (c/L)		Total Protein Values 56.9–78.7 (g/L)	
	March 2020	July 2020	March 2020	July 2020	March 2020	July 2020
**C1**	0.32	0.32	22.95	11.30	82	72
**C2**	0.30	0.30	24.00	11.65	72	62
**C3**	0.30	0.30	24.40	10.25	76	64
**C4**	0.30	0.30	7.80	8.00	74	70
**C5**	0.39	0.39	24.00	10.80	62	58
**C6**	0.32	0.32	9.95	8.05	76	70
**C7**	0.30	0.30	12.05	11.00	58	74
**C8**	0.30	0.30	11.65	10.95	73	68

* Hematocrit (HCT); white blood cells (WBCs).

**Table 2:** Vector-borne pathogens detected in blood samples collected from cattle using real-time microfluidic PCR.

Vector-Borne Pathogen(s)	Total	%	95% CI ^a^
**Total infected samples (≥1 pathogen)**	**32**	**100**	95–100
*A. marginale*	31	96.9	90.9–100
*R. felis*	4	12.5	1.10–23.9
*Rickettsia* spp.	2	6.25	0–7.09
*Anaplasma* spp.	1	3.12	0–9.19
**Single infections**	**26**	**81.3**	**67.8–94.8**
*A. marginale*	26	81.3	67.8–94.8
**Mixed infections**	**6**	**18.8**	**5.3–32.5**
*R. felis* + *A. marginale*	3	9.38	0–19.5
*A. marginale*+ *Rickettsia* spp.	2	6.25	0–7.09
*R. felis*+ *Anaplasma* spp.	1	3.13	0–0.11

^a^ 95% confidence interval.

**Table 3:** Vector-borne pathogens detected in ticks collected from cattle using real-time microfluidic PCR.

Vector-Borne Pathogen(s)	Total	%	95% CI ^a^
**Total infected ticks (≥1 pathogen)**	**15**	**62.5**	**60.6–64.4**
*A. marginale*	11	45.8	25.9–65.7
*R. felis*	4	16.7	1.81–31.5
*Rickettsia* spp.	1	4.17	0.1–8.24
*Ehrlichia* spp.	2	8.33	2.69–13.9
**Single infections**	**12**	**50.0**	**41.4–58.6**
*A. marginale*	8	33.3	14.2–51.8
*R. felis*	3	12.5	5.75–19.3
*Ehrlichia* spp.	1	4.17	0.1–8.24
**Mixed infections**	**3**	**12.5**	**5.75–19.3**
*A. marginale* + *Rickettsia* spp.	1	4.17	0.1–8.24
*A. marginale* + *Rickettsia felis*	1	4.17	0.1–8.24
*B. canis* (subspecies) + *A. marginale* + *Ehrlichia* spp.	1	4.17	0.1–8.24

^a^ 95% confidence interval.

## Data Availability

DNA sequences obtained in this study were submitted to GenBank (https://www.ncbi.nlm.nih.gov, accessed on 05 March 2023), and accession numbers were assigned (OQ362275-OQ362281, OQ619389-OQ619392).

## Supplementary Materials

The following supporting information can be downloaded at: https://www.mdpi.com/article/10.3390/pathogens12080998/s1, Table S1. List of primer/probe sets used in the BioMark™ real-time PCR system.
